# Supplementary figures and images for: Heparan sulfate-dependent RAGE oligomerization is indispensable for pathophysiological functions of RAGE
Source: eLife. 2022 Feb 9;11:e71403. doi: 10.7554/eLife.71403 (PMC8863369; doi:10.7554/eLife.71403)

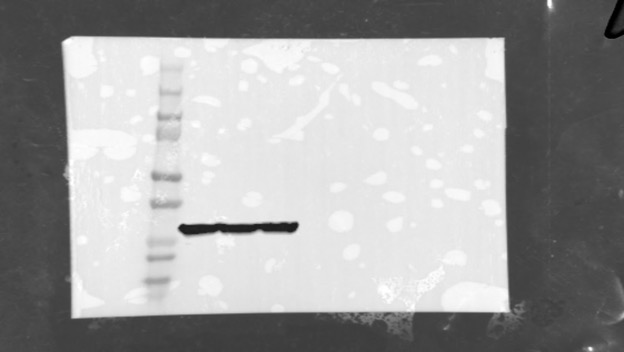

Supplement: Figure 1—source data 2. [file elife-71403-fig1-data2.zip › Figure_1-_source_data_2/Figure 1-source data 2-raw blot-2.jpg]

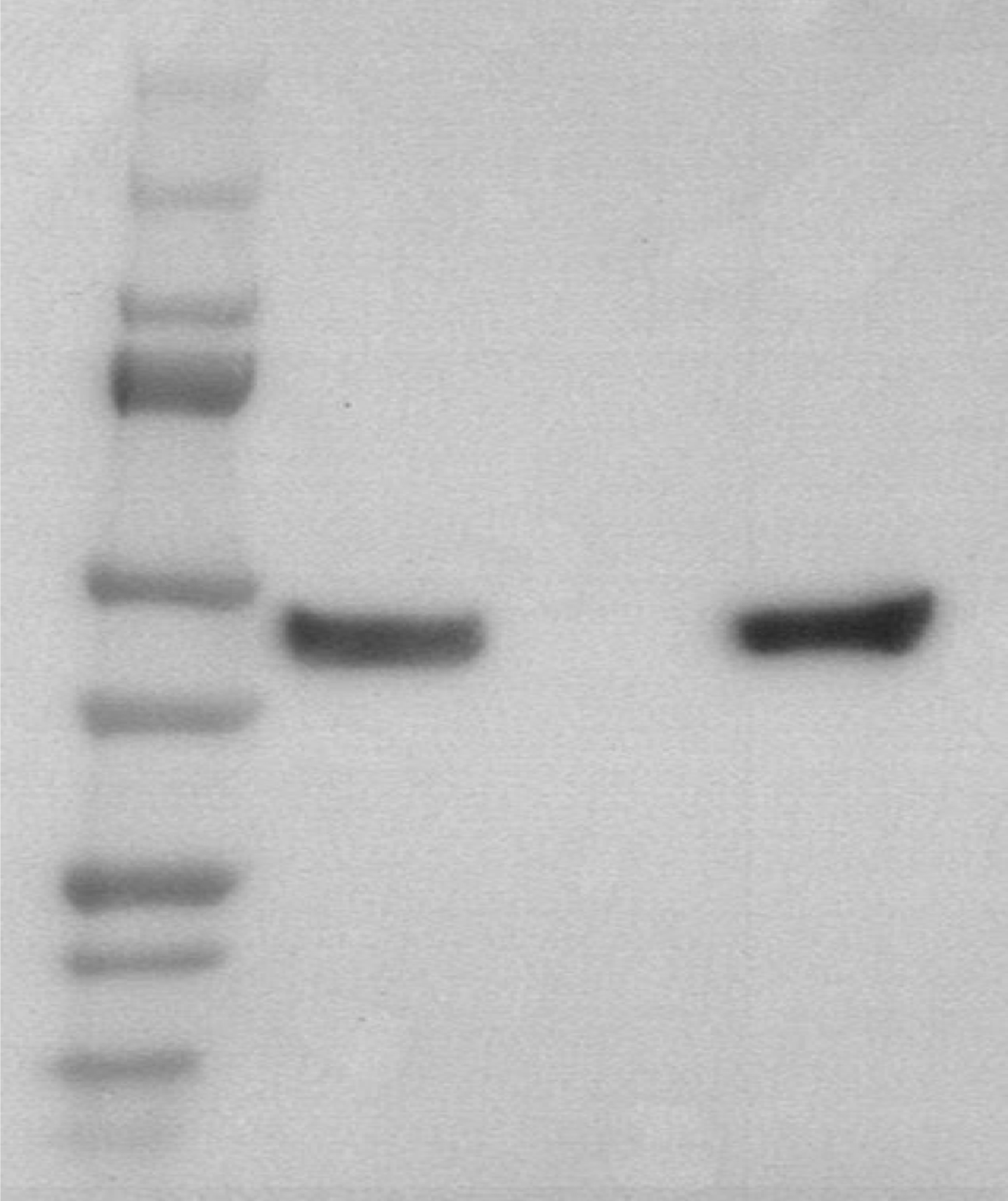

Supplement: Figure 1—source data 2. [file elife-71403-fig1-data2.zip › Figure_1-_source_data_2/Figure 1-source data 2-raw blot-1.png]

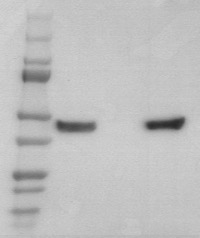

Supplement: Figure 1—source data 2. [file elife-71403-fig1-data2.zip › Figure_1-_source_data_2/Figure 1-source data 2-raw blot-1.jpg]

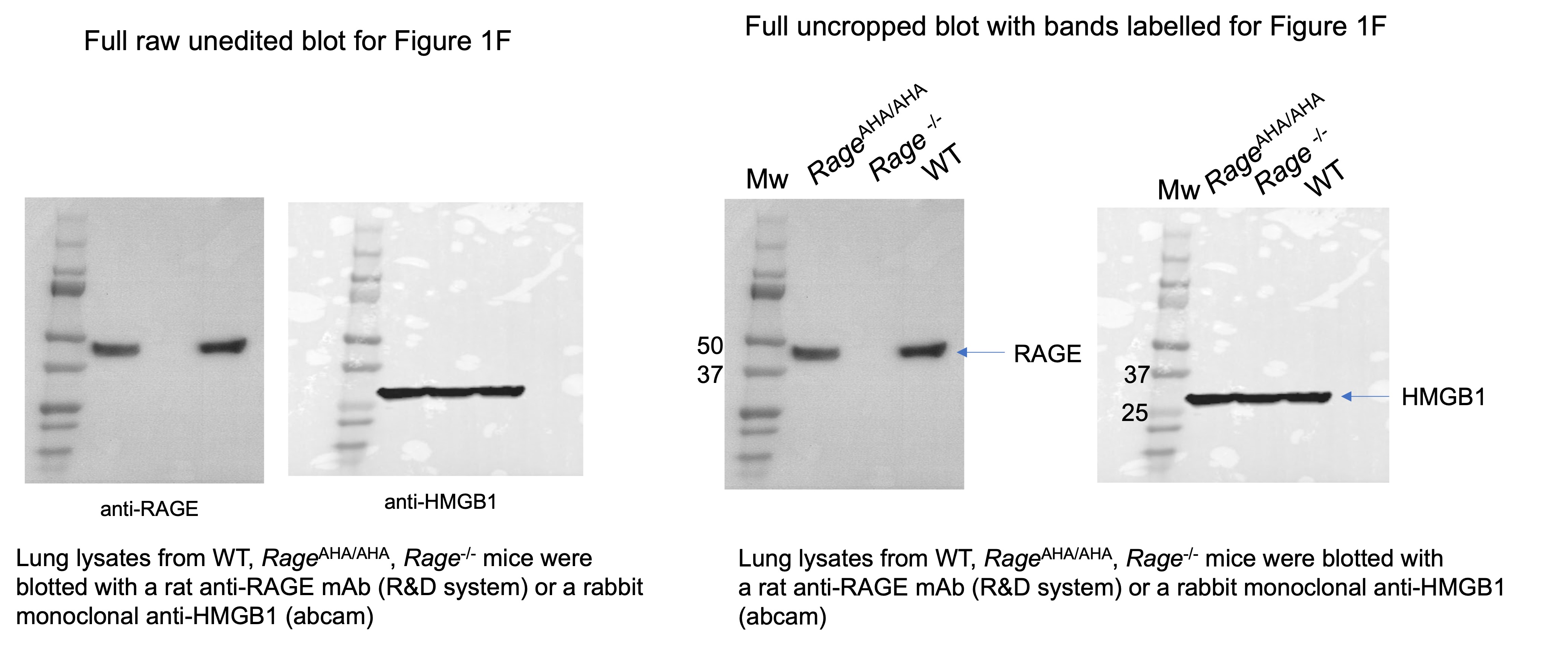

Supplement: Figure 1—source data 2. [file elife-71403-fig1-data2.zip › Figure_1-_source_data_2/Figure 1- source data 2.jpg]

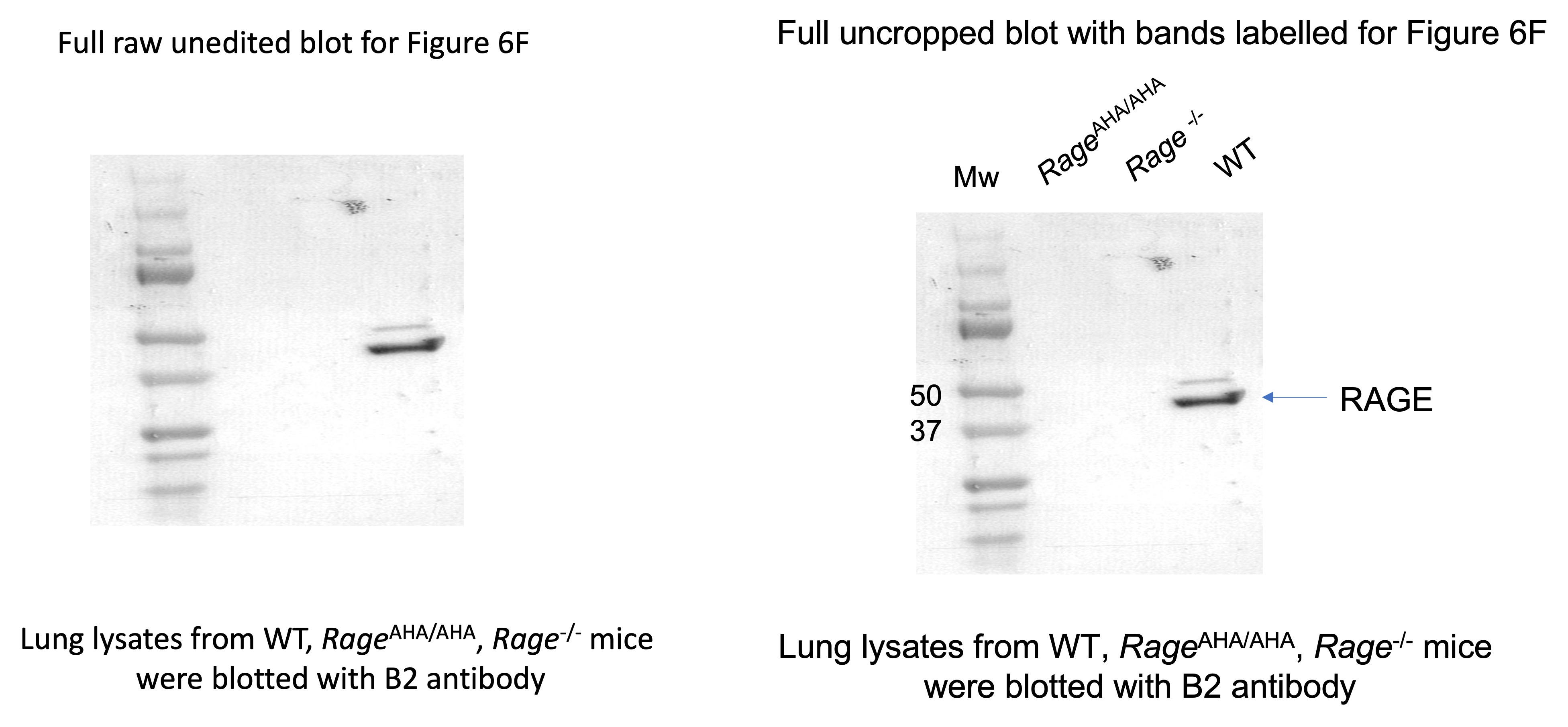

Supplement: Figure 6—source data 2. [file elife-71403-fig6-data2.zip › Figure_6-source_data_2/Figure 6-source data 2.jpg]

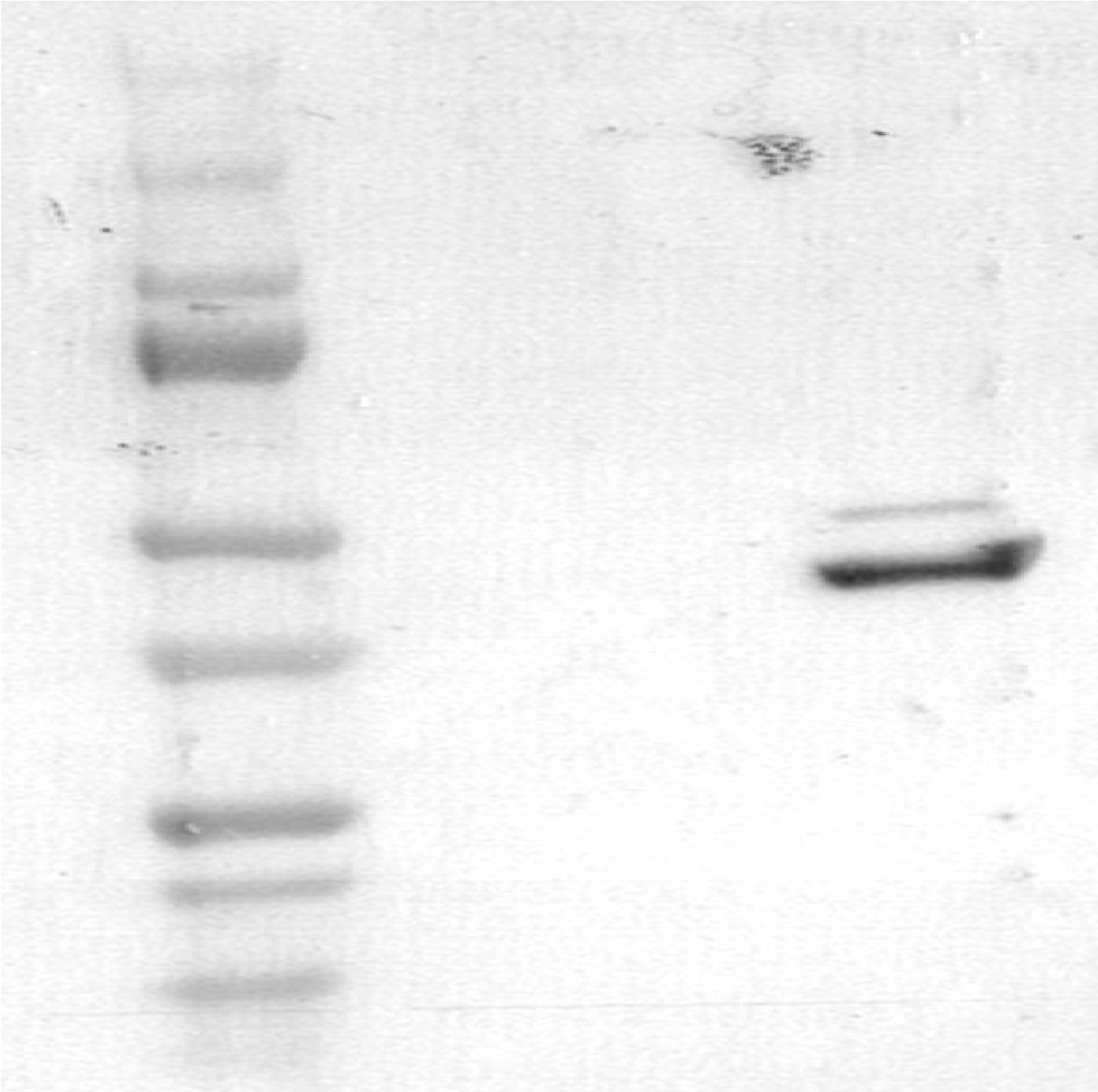

Supplement: Figure 6—source data 2. [file elife-71403-fig6-data2.zip › Figure_6-source_data_2/Figure 6-source data 2-raw blot.png]

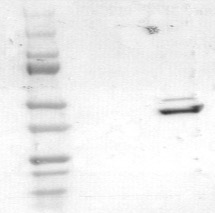

Supplement: Figure 6—source data 2. [file elife-71403-fig6-data2.zip › Figure_6-source_data_2/Figure 6-source data 2-raw blot.jpg]
